# Gardening for Health: Using Garden Coordinators and Volunteers to Implement Rural School and Community Gardens

**DOI:** 10.5888/pcd16.190117

**Published:** 2019-11-27

**Authors:** Suzanne Stluka, Lacey A. McCormack, Linda Burdette, Samantha Dvorak, Nathania Knight, Rachel Lindvall, Lauren Pierce, Jason Schoch, Prairey Walkling

**Affiliations:** 1 Extension, Montana State University, Bozeman, Montana; 2 Health and Nutritional Sciences Department, South Dakota State University, Brookings, South Dakota; 3 Undergraduate Nursing, South Dakota State University, Brookings, South Dakota; 4 Extension, South Dakota State University, Brookings, South Dakota

## Abstract

Gardens provide access to healthy food, increase access to nutrition and physical activity opportunities, and are a focal point for community interventions. We used a gardening intervention to improve local access to and consumption of fruits and vegetables and as an integral part of overall efforts of local wellness coalitions. Seasonal garden coordinators were hired, and action plans included goals for nutrition and physical activity education programs and youth and adult engagement. The characteristics of each garden (size, items planted, number of volunteers) and pre- and post-intervention surveys were used to understand how the gardens affected communities. Thirteen gardens were planted, and volunteers provided 18,136 hours; adults from the community reported an increased awareness of garden benefits. The community garden intervention provided opportunities for collaboration with a variety of schools, community organizations, and city and tribal organizations, thereby increasing the sustainability of the intervention.

**Summary**

**What is already known about this topic?**

Several states participated in the Centers for Disease Control and Prevention High Obesity grant project. Community gardens increase access to and availability of healthy food, increase physical activity of gardeners, and provide numerous social and emotional benefits.

**What is added by this report?**

From 2014 through 2018 community gardens were established across 6 counties in rural South Dakota with the goal of engaging community members to improve food access and resources and be more physically active. We describe this process.

**What are the implications for public health practice?**

Gardens are a part of the local foods landscape in rural communities; thus, understanding how community gardens can influence broad community policies, systems, and environments can help other communities to develop and implement similar programs.

## Background

Community gardens increase access to and availability of healthy food (1), and fruit and vegetable consumption is higher among adults who participate in community garden projects than those who do not, in both rural (2) and urban areas (3). Physical activity levels are also higher among community gardeners, because the work done in gardens constitutes moderate-to-high physical activity (4). Furthermore, people who garden have lower body mass indexes than those who do not (5), probably because of the diet- and physical activity–related benefits of gardens and gardening.

Connecting and interacting with nature itself has many health benefits (6), and the benefits of community gardens go beyond improving diet, physical activity, and weight outcomes. Numerous social and emotional benefits of community gardening have been documented, including social interaction (7), strengthened family relationships (8), community building and engagement (3,7,9), and greater life satisfaction (9). Moreover, food insecurity has been reduced in certain populations by community gardening (8), which also has been shown to increase food self-sufficiency (10,11). Although community gardening efforts have many benefits of their own, gardens themselves can also serve as a focal point for other interventions in the community, including family gatherings, community meetings, and improving physical and mental well-being (12).

Communities and academic entities can successfully partner to develop community gardens (13), which are a vital part of a healthy community approach and are built-in aspects of the community engagement process. South Dakota State University (SDSU) Extension worked with local communities to implement garden-based interventions that were tailored to meet the needs of rural South Dakotans, including tribal communities. The purpose of the gardens was to improve local access to and consumption of fruits and vegetables in counties with prevalence of adult obesity higher than 40% and with a high percentage of Supplemental Nutrition Assistance Program (SNAP) participants. We describe how community garden coordinators and volunteers were used to implement school and community gardens and the public health implications of the project.

## Partnerships and Collaborations

SDSU Extension staff members and collaborators first engaged and supported communities in establishing a local wellness coalition as part of the Centers for Disease Control and Prevention’s (CDC’s) High Obesity Program and the Supplemental Nutrition Assistance Program Education’s (SNAP-Ed’s) cooperative agreements to empower communities to implement environmental interventions, such as gardens. The newly formed coalitions recruited and engaged community members and raised awareness about coalition efforts. Each coalition had an Extension staff member who acted as lead facilitator and helped to ensure that activities kept moving forward. Local community wellness coalitions met monthly, and some more frequently, depending on the project they were working on. All wellness coalitions were asked to create a food and demonstration garden. The overall intent of these gardens was to demonstrate a holistic approach that consisted of nutrition education, access to healthy foods, gardening instruction, and physical activity to make gardening and related activities an integral part of the overall efforts of the local wellness coalitions.

## Implementing Rural and Community Gardens

Each community wellness coalition decided where the gardens would be located on the basis of access to resources, management, and other factors. Existing gardens were acceptable as long as they were welcoming and open to all members of the community and had appropriate site management strategies in place. Once the wellness coalition chose its garden sites, a garden action plan was created for each site to determine the intended scope of the project (eg, type of crops, planting strategies, growing structures such as raised beds, access to water). All garden sites were to include youth and adult engagement in planting and growing food, and harvesting, processing, and preserving produce. The wellness coalitions set progressive goals for nutrition and physical activity education programs and the size and scope of the garden each year. Many of the garden sites incorporated policy and system changes as a result of the development and maintenance of the gardens. For example, policy aspects included shared use agreements and zoning, and systems changes included composting efforts and access to water.

Seasonal garden coordinators were hired from within the communities and were trained to assist each community with their gardening efforts, such as developing new gardens, assisting with current gardening efforts, and helping to build high tunnel systems (unheated greenhouses that can help farmers extend their growing season). The garden coordinators staffing model was different for each community. Most communities hired 2 garden coordinators to distribute the work. The garden coordinators worked from April through September, and each community was allowed up to 520 hours of work by the garden coordinators. The overall responsibilities of the garden coordinators were to assist community members with gardening, encourage kids and adults to garden while growing food for their local community, recruit community members to participate in gardening and nutrition education activities, train volunteer groups to assist with gardens and provide nutrition and physical activity lessons at the garden, and maintain garden records. Garden coordinators were trained by SDSU Extension before the start of their employment, and many were current SDSU Extension Master Gardeners with over 8 weeks of previous garden training experience (14).

The goal of the project was to provide garden coordinators with resources and simple, easy-to-use tracking tools for successful implementation of the project. The garden-characteristic tool provided information on garden size, number of plots, location of water, results of soil testing, and types of food produced. The produce-tracking tool provided information on types of produce, number of items harvested, whether produce was donated (eg, to a food pantry), sold, or used by volunteers and their families. Produce lost to spoilage and theft were estimated. The garden visitor log tracked volunteers (youth and adult), distance traveled, activities completed, and estimated time spent at the garden. Garden coordinators also tracked their time spent working at the garden by using a detailed time sheet submitted monthly as part of the payroll process. Additionally, nutrition education was conducted at the garden sites for both youth and adults as a part of SNAP-Ed outreach efforts.

## Benefits of Community Gardens in Schools and Communities

All communities created community action plans and budgets for community garden implementation. The average garden size across all sites was 1,495 square feet (range, 32 square feet to 4,362 square feet). Gardens used plots, raised beds, and tire beds. Each individual garden harvested an average of 138 pounds and 232 items. One garden harvested 770 pounds of produce. Most produce was donated to food pantries and volunteers. One site donated 75% of its produce to the YMCA feeding program in its community. From May 2017 through September 2017, garden volunteers provided 18,136 hours in garden management. This represents a value of $386,297 in service to South Dakota communities (15). Volunteers traveled various distances. In one community, volunteers traveled an average of one-half mile to work at the garden. In a more rural community, volunteers traveled an average of 8 miles. Some outlier volunteers traveled over 200 miles round trip.

**Figure 1:**
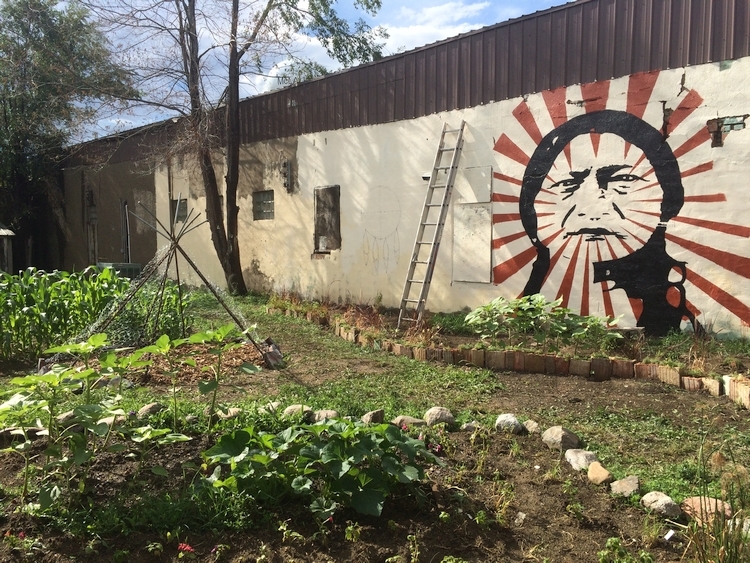
The Standing Rock Boys and Girls Club started with a simple grow station and then transitioned their garden outdoors to create the Wakanyeja “Beginning of Life” garden where all are welcome.

**Figure 2:**
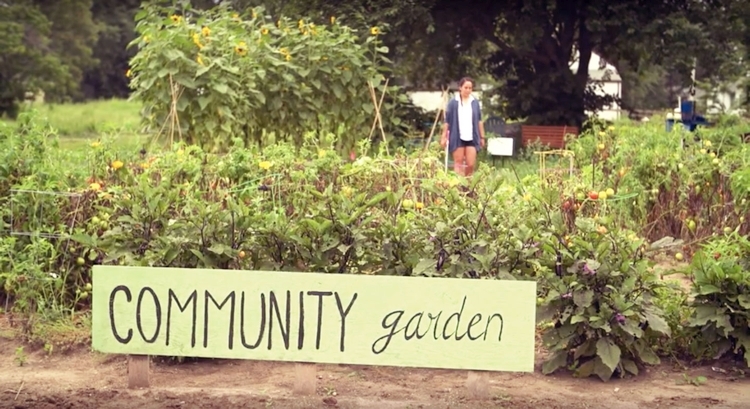
The Marty Boys and Girls Club developed a video, Wakaniza Ta'owozupi, Children’s Garden video, entitled *Growing Healthy Food, Families, and Communities Across South Dakota.* https://www.youtube.com/watch?v=QgbSPRH2WB8.

In one tribal community, the wellness coalition collaborated with a local summer school to plant a raised box garden of yellow crookneck squash. The students then participated in the “Grow it, Try it, Like it” SNAP-Ed nutrition education program. Another tribal community planted and harvested sweetgrass and sage. A portion of the sage was donated to the local school for morning smudging. The Standing Rock Boys and Girls Club started with a simple grow station and then transitioned their garden outdoors to create the Wakanyeja “Beginning of Life” Garden (Figure 1). The Marty Boys and Girls Club initiated Wakaniza Ta'owozupi “Children’s Garden,” described in their video *Growing Healthy Food, Families and Communities Across South Dakota* (Figure 2), which has expanded to include a high tunnel, to use produce for healthy snacks. All used innovative gardening techniques to extend the growing season while providing a neighborhood gathering place.

## Implications for Public Health

Working with communities to develop community gardens is a large undertaking, and external funding helped to kick-start these efforts. Dedicated, trained seasonal garden coordinators made the maintenance and sustainability of the gardens possible, and selecting coordinators from within the community helped to quickly establish trust and buy-in from other community members. Although garden coordinators received training before their start and had access to trained SDSU Extension staff members throughout their employment, we strongly recommend that the position of garden coordinator require completion of Master Gardening training (14). Furthermore, to engage the community, we recommend that previous experience and passion for the job be considered. 

Few garden tracking evaluation tools were applicable to measuring project outcomes; therefore, in the communities, we modified and piloted examples found through a review of the literature. Evaluation tools used at the gardens had to be easily completed by community members, because extension staff members were not always present for data collection. Scales to weigh the produce also had to be easy to use and had to withstand changes in weather throughout the garden season because they remained outside in the elements. Furthermore, a lockbox system was also needed that could withstand the weather and protect data collection. Using garden coordinators to distribute and collect surveys may increase efficiency.

The community garden intervention provided opportunities for collaboration with a variety of schools, community organizations, and city and tribal organizations. In one tribal community, the city provided water as an in-kind donation, and the local YMCA provided garden space. Another tribal community developed an agreement with the Indian Health Service for water use. This collaboration and leveraging of funds will support sustainability of the community gardens.

Gardens are a part of the local food landscape in rural communities, which face limited food access and high rates of food insecurity. This project showed that community gardens can produce substantial amounts of produce, as evidenced by the 770 pounds of produce grown in one garden. In addition, community members were willing to contribute volunteer hours to the success and sustainability of the gardens. However, further exploration into what groups in these rural communities are using the produce, how they are using it, and its effect on diet quality and food security is still needed. Furthermore, the ability of gardens to influence broad community policies, systems, and environments, such as integration into farmers markets and farm-to-school, still need to be explored.
